# Reporting Guidelines and Issues to Consider for Using Intracranial Brain Stimulation in Studies of Human Declarative Memory

**DOI:** 10.3389/fnins.2018.00905

**Published:** 2018-12-04

**Authors:** Nanthia Suthana, Zahra M. Aghajan, Emily A. Mankin, Andy Lin

**Affiliations:** ^1^Department of Psychiatry and Biobehavioral Sciences, David Geffen School of Medicine, Jane and Terry Semel Institute for Neuroscience and Human Behavior, UCLA, Los Angeles, CA, United States; ^2^Department of Neurosurgery, David Geffen School of Medicine, UCLA, Los Angeles, CA, United States; ^3^UCLA, Los Angeles, CA, United States; ^4^IDRE Statistical Consulting Group, UCLA, Los Angeles, CA, United States

**Keywords:** declarative memory, intracranial stimulation, deep brain stimulation, humans, medial temporal lobe

## Abstract

Participants with stimulating and recording electrodes implanted within the brain for clinical evaluation and treatment provide a rare opportunity to unravel the neuronal correlates of human memory, as well as offer potential for modulation of behavior. Recent intracranial stimulation studies of memory have been inconsistent in methodologies employed and reported conclusions, which renders generalizations and construction of a framework impossible. In an effort to unify future study efforts and enable larger meta-analyses we propose in this mini-review a set of guidelines to consider when pursuing intracranial stimulation studies of human declarative memory and summarize details reported by previous relevant studies. We present technical and safety issues to consider when undertaking such studies and a checklist for researchers and clinicians to use for guidance when reporting results, including targeting, placement, and localization of electrodes, behavioral task design, stimulation and electrophysiological recording methods, details of participants, and statistical analyses. We hope that, as research in invasive stimulation of human declarative memory further progresses, these reporting guidelines will aid in setting standards for multicenter studies, in comparison of findings across studies, and in study replications.

## Introduction

The use of surgically implanted electrodes within the human brain has become increasingly common in treating and/or evaluating abnormal brain activity in patients with epilepsy (Schulze-Bonhage, 2017), Parkinson's disease (Benabid et al., 1987), and dystonia (Vidailhet et al., 2005) and is also being explored in depression (Ressler and Mayberg, 2007), obsessive compulsive disorder (Nuttin et al., 1999), and Alzheimer's disease (Lozano et al., 2016). With a continued rise in medical treatments using implanted neural devices, research opportunities to record from, and stimulate, the brain during human cognition will also likely increase. For the study of human declarative memory, it is common to work with participants with temporal lobe epilepsy (TLE), as the temporal lobe plays an important role in forming and retrieving declarative memories (i.e., facts and events). In TLE, seizures are thought to originate from a site within the temporal lobe, and electrodes are therefore either placed on the surface—such as in lateral temporal cortex—or implanted deeply within medial temporal lobe (MTL) regions, making research studies on declarative memory modulation using direct brain stimulation possible.

TLE patients with implanted electrodes are relatively rare in a research setting, and thus single-site intracranial stimulation studies of declarative memory frequently suffer from low sample sizes (e.g., < 10). Even if multi-site studies can claim large samples (e.g., >20) through data collection at multiple surgical centers, numerous factors such as electrode placement, characteristics (e.g., diameter) and implantation approaches often vary within these samples, resulting in small homogeneous subsamples used for statistical analyses. An important goal of these studies should therefore be to report findings in a way that will allow for accurate evaluation, replication, and future meta-analyses. Since the use of intracranial stimulation for memory modulation is still a relatively small field, the goal of this mini-review is to provide a set of reporting guidelines that will facilitate future comparisons across studies and proper replication. We hope these guidelines will generate productive discussions between fellow researchers and provide a framework to guide design and/or evaluation of similar studies. Similar efforts have proven to be productive in other research fields such as functional magnetic resonance imaging (Poldrack et al., 2008). A summary of relevant studies is presented in Table 1, guidelines are discussed below and a reference checklist is provided in Appendix A.

**Table 1:** Summary of intracranial stimulation studies of declarative memory.

**Study**	**Type of memory**	**Memory impairment or improvement**	**Area(s) of stimulation**	**Unilateral/Bilateral**	**Electrode type**	**Electrode properties**			**Electrode implantation approach**
						**Diameter**	**Length**	**Spacing**	
Halgren et al., 1985	Complex scenes (recognition)	Impairment	MTL	Bilateral	Depth	NR	NR	~1 mm	NR
Halgren and Wilson, 1985	Word pairs (recall)	Impairment	MTL	Unilateral	Depth	NR	NR	~1 mm	NR
Fernández et al., 1996	Words	Impairment	Hippocampus	Unilateral	Depth	NR	NR	37 mm	Transoccipital
					Subdural	NR	NR	Variable	NA
Coleshill et al., 2004	Words, objects, faces (recognition)	Impairment	Hippocampus	Unilateral	Depth	NR	NR	NR	NR
Hamani et al., 2008	Word pairs (recognition)	Improvement	Fornix	Bilateral	Depth	1.27 mm	1.5 mm	1.5 mm	NR
Lacruz et al., 2010	Words, objects, faces (recognition)	Impairment	Hippocampus	Bilateral	Depth	NR	2.3 mm	5 mm	Temporolateral
Fell et al., 2013	Words (recall and recognition)	Improvement (trend)	Rhinal cortex and hippocampus	Bilateral	Depth	1.3 mm	1.6 mm	1.5 mm	Variable
Suthana et al., 2012	Spatial navigation	Improvement	Entorhinal	Unilateral	Depth	1.5 mm	1.5 mm	1.5 mm	Temporolateral
Koubeissi et al., 2013	Mini-mental status exam	Improvement	Fornix	Unilateral	Depth	1.1 mm	2.3 mm	5 mm	NR
Miller et al., 2015	Complex figures (recall)	Improvement	Fornix	Unilateral	Depth	1.1 mm	2.3 mm	5 mm	Temporolateral
Jacobs et al., 2016	Words (recall), spatial navigation	Impairment	MTL	Unilateral	Depth	area = 0.059 cm^∧2^	NR	NR	Variable
					Subdural	NR	NA	NR	NA
Titiz et al., 2017	Persons (recognition)	Improvement	Entorhinal	Unilateral	Depth	100 μm	NA	NA	Temporolateral
Merkow et al., 2017	Words (recall)	Impairment	MTL	Unilateral	Depth	1.2 mm	2.4 mm	8 mm	NR
					Subdural	2.4mm	NA	10 mm	NA
Ezzyat et al., 2017	Words (recall)	Improvement/Impairment	Variable	Unilateral	Depth	NR	NR	5 mm	Variable
					Subdural	NR	NR	10 mm	NA
Kucewicz et al., 2018a	Words (recall)	Improvement	Lateral temporal	Unilateral	Depth	NR	NR	1.5–10 mm	Variable
					Subdural	NR	NR	10 mm	NA
Kucewicz et al., 2018b	Words (recall)	Improvement	Lateral temporal	Unilateral	Depth	1–10 mm	NR	5–10 mm	Variable
					Subdural	1–10 mm	NR	10 mm	NA
Ezzyat et al., 2018	Words (recall)	Improvement	Lateral temporal	Unilateral	Depth	0.8–2.3 mm	NR	NR	Variable
					Subdural	0.8–2.3 mm	NR	NR	NA
Kim et al., 2018	Spatial navigation, temporal order	Impairment (spatial); no effect (temporal)	Variable	Unilateral	Depth	0.8 mm	2.5 mm	3–3.75 mm	NR
Hansen et al., 2017	Word-color pairs (recognition)	No effect	Entorhinal	Unilateral	Depth	1.3 mm	1.6 mm	3 mm, 4.5 mm	Temporolateral
Inman et al., 2018	Objects (recognition)	Improvement	Amygdala	Unilateral	Depth	0.86 mm	2 mm	5 mm	NR
Halgren et al., 1985	Bipolar	< 1.5 mA	100 μs	Single Pulse	< 17 μC	2–8.3 KΩ	Stimulus (encoding)	Altered LFP and spiking	NR	NR
Halgren and Wilson, 1985	Bipolar	< 2 mA	100 μs	Variable	< 22 μC	2–8.3 KΩ	Stimulus (encoding)	Afterdischarges	NR	NR
Fernández et al., 1996	Bipolar	3 V	2.5 ms	2 Hz	1.08–8.30 μC	NR	Continuous	NA	NR	R
									
Coleshill et al., 2004	Bipolar	NR	1 ms	50 Hz	57 μC	NR	Stimulus (encoding)	NA	R	NR
Hamani et al., 2008	Bipolar	3–5 V	60 μs	130 Hz	NR	NR	Continuous	Increased metabolic activity	NA	NA
Lacruz et al., 2010	Bipolar	4–6 mA	1 ms	Single pulse	NR	NR	Stimulus (encoding and retrieval)	NA	NR	R
Fell et al., 2013	Bipolar	0.01 mA	NR	40 Hz	< 1.25 μC	5–25 KΩ	Continuous	Gamma phase synchrony	NR	R
Suthana et al., 2012	Bipolar	0.5–1.5 mA	300 μs	50 Hz	2.5–7.6 μC	1–4 KΩ	5 s on/off	Theta phase resetting	R	R
Koubeissi et al., 2013	Bipolar	8 mA	0.3 ms	5 Hz	20 μC	NR	Continuous	Evoked responses	>2 days prior	R
Miller et al., 2015	Bipolar	7 mA	0.1 ms	Theta burst (200 Hz at 5 Hz, 1 s)	NR	NR	Continuous	Diffused evoked potential	NR	NR
Jacobs et al., 2016	Bipolar	0.5–1.5 mA	300 μs	50 Hz	NR	NR	Pre-stimulus (encoding)	NA	NR	NR
		< 3 mA		50 Hz					
Titiz et al., 2017	Monopolar	150 μA	200 μs	Theta burst (100 Hz at 5 Hz, 1 s)	9.32 μC	< 60 KΩ	Pre-stimulus (encoding)	NA	R	R
Merkow et al., 2017	Bipolar	1.9–5.5 mA	300 μs	50 Hz	6.8–39.8 μC	NR	5 s (distraction)	NA	NR	R
									
Ezzyat et al., 2018a	Bipolar	< 1.5 mA	300 μs	50 Hz	NR	NR	Stimulus (encoding)	Increased high and decreased low frequency power	NR	R (hemisphere)
		< 3.5 mA							
Kucewicz et al., 2018a	Bipolar	< 1.5 mA	300 μs	50 Hz	NR	NR	Stimulus (encoding)	High gamma activity	NR	R
		< 3.5 mA							
Kucewicz et al., 2018b	Bipolar	< 1.5 mA	300 μs	50 Hz	NR	NR	Stimulus (encoding)	Increased gamma power	NR	R
		< 3.5 mA							
Ezzyat et al., 2018	Bipolar	< 1.5 mA	300 μs	Variable	NR	NR	Stimulus (encoding)	Increase in classifier output	NR	R (hemisphere)
		< 3.5 mA							
Kim et al., 2018	Bipolar	4–5 mA	500 μs	Theta burst (50 Hz at 4 Hz, 2 s)	NR	NR	Inter-trial-interval (retrieval)	Reduced theta phase connectivity	NR	R
Hansen et al., 2017	Bipolar	0.1 mA; < 1V	300 μs	50 Hz	< 0.5 μC	< 10 KΩ	15 s on/off (encoding)	Positive event-related potentials	NR	R
Inman et al., 2018	Bipolar	1–12 V (1–15 mA)	300 μs	Theta burst (50 Hz at 8 Hz, 1 s)	NR	0.8–110 KΩ	Post-stimulus (encoding)	Theta gamma coupling	R	R

*Summarized are studies that have utilized intracranial stimulation as a method to modulate human hippocampal dependent declarative memory. Included are the types of memory tasks used, what behavioral effects (improvement or impairment) were found, brain areas stimulated, whether stimulation was bilateral or unilateral, the type of electrode used (i.e., depth or subdural: strip ,or grid), electrode properties (i.e., diameter and length of electrode contacts and spacing between stimulated contacts), surgical approach of implantation, and specific stimulation parameters used such as bi/mono-polar, current amplitude, frequency of stimulation, pulse width (pw), train duration, charge density (μC/cm2/phase), impedance values, during what phase of the task or trial stimulation was given (stimulation timing) if reported. Lastly, neurophysiological changes, if investigated, are summarized. For further detailed discussion of these studies see previous reviews Suthana and Fried, 2014; Kim et al., 2016; Sreekumar et al., 2017. MTL, medial temporal lobe; PW, pulse width; R, Reported; NR, Not Reported; NA, Not Applicable; AED, antiepileptic drugs; and SOZ, seizure onset zone. Stimulation amplitude is reported in current (A) or voltage (V). Charge density values are reported per cm2/phase*.

## Describe the Participants Fully

The ability to probe the human brain and apply electrical stimulation to investigate cognitive functions is a unique and an invaluable opportunity. It must be noted, however, that these experiments are largely done in patients with epilepsy and consequently, there is a concern regarding the generalizability of results to the normal population. Thus, when interpreting the results, there are certain confounding factors that cannot be eliminated but must be explicitly mentioned and taken into consideration.

Furthermore, because this data acquisition is often difficult and time consuming, published findings usually have few participants within a given statistical group. To facilitate comparisons across studies and ease their integration into future larger meta-analyses, detailed information of study participants should be provided. In addition to basic demographic information, studies should report relevant clinical information such as medications, neuropsychological scores, MRI abnormalities, comorbid neuropsychiatric conditions, and determined seizure onset zone (SOZ). It has been shown that interictal epileptic activity can interfere with cognitive performance (Kleen et al., 2013; Ung et al., 2017), so when possible, a quantification of the frequency of such events should be included.

One important consideration is that the targeting of implanted electrodes is determined based on clinical criteria. This implies that some implanted electrodes, potentially including the stimulating electrode, can fall within the SOZ. Stimulating the SOZ can lead to afterdischarges or other nonspecific effects (Cherlow et al., 1977; Kesner, 1982; Halgren and Wilson, 1985) that can interfere with results. It is, thus, necessary to be cognizant of any interactions between stimulation effects on memory and seizure activity. If stimulating the SOZ cannot be avoided, one possibility is to compute statistical tests using data from all stimulating electrodes, and then confirm whether results are consistent when considering only data from when the stimulating electrode was in clinically determined “healthy tissue.” A discrepancy could indicate that stimulation in the SOZ differentially affects memory processes. Any relationship between memory effects and seizure activity or proximity of electrode to SOZs should be reported. At minimum, the determined SOZ, occurrence of seizures, and/or seizure related activity should be reported for each participant.

Yet another factor in conducting memory research in epilepsy patients is that participants can have impaired cognitive functions. This can potentially influence the efficacy of stimulation on memory modulation. Thus, it is important to include participant level information about cognitive abilities. Ideally, results reported will be pertinent to the type of memory tested with stimulation. Examples of relevant neuropsychological tests reported in previous studies include: Wechsler Memory Scale (verbal memory) (Wechsler, 2005); California Verbal Learning Test (verbal memory) (Delis et al., 2000); Rey–Osterrieth Complex Figure Test (visual memory) (Meyers and Meyers, 1995).

Clinical studies using DBS in Parkinson's disease have highlighted the effects medication can have on neurophysiological activity and responsiveness to stimulation (Brown and Williams, 2005). While clinical circumstances in epilepsy patients do not allow for researchers to control the presence of medication, studies should report details of medications that participants take, including the type of medication and, ideally, the time of most-recent administration relative to study completion.

## Report in Detail on Electrode Characteristics and Electrode Localization Methods

Recent clinical DBS studies have emphasized the importance of precise electrode location in treatment outcome. The precise position of electrodes within white or gray matter could be critical for efficacy of treatment in both Parkinson's disease and depression (Pouratian et al., 2011; Riva-Posse et al., 2014). Implanted electrodes in TLE patients are targeted to specific regions based on clinically hypothesized SOZs in each individual. Therefore, studies often contain substantial variability among electrode locations. While subdural (i.e., strip and grid) electrodes lie on the surface of the brain and affect gray matter areas, depth electrodes can stimulate white and/or gray matter, which can have different effects on behavioral outcomes (Titiz et al., 2017). Furthermore, areas like the amygdala consist of distinct nuclei where stimulation location may be critical (Inman et al., 2018). Providing high-resolution MRI and DTI would allow for the reporting of electrode locations within specific gray matter subregions and/or white matter pathways.

Studies should report in detail how electrode contact locations are determined; namely, the type of registration procedure used and how electrode contacts and brain regions are visualized.

**Figure 1:**
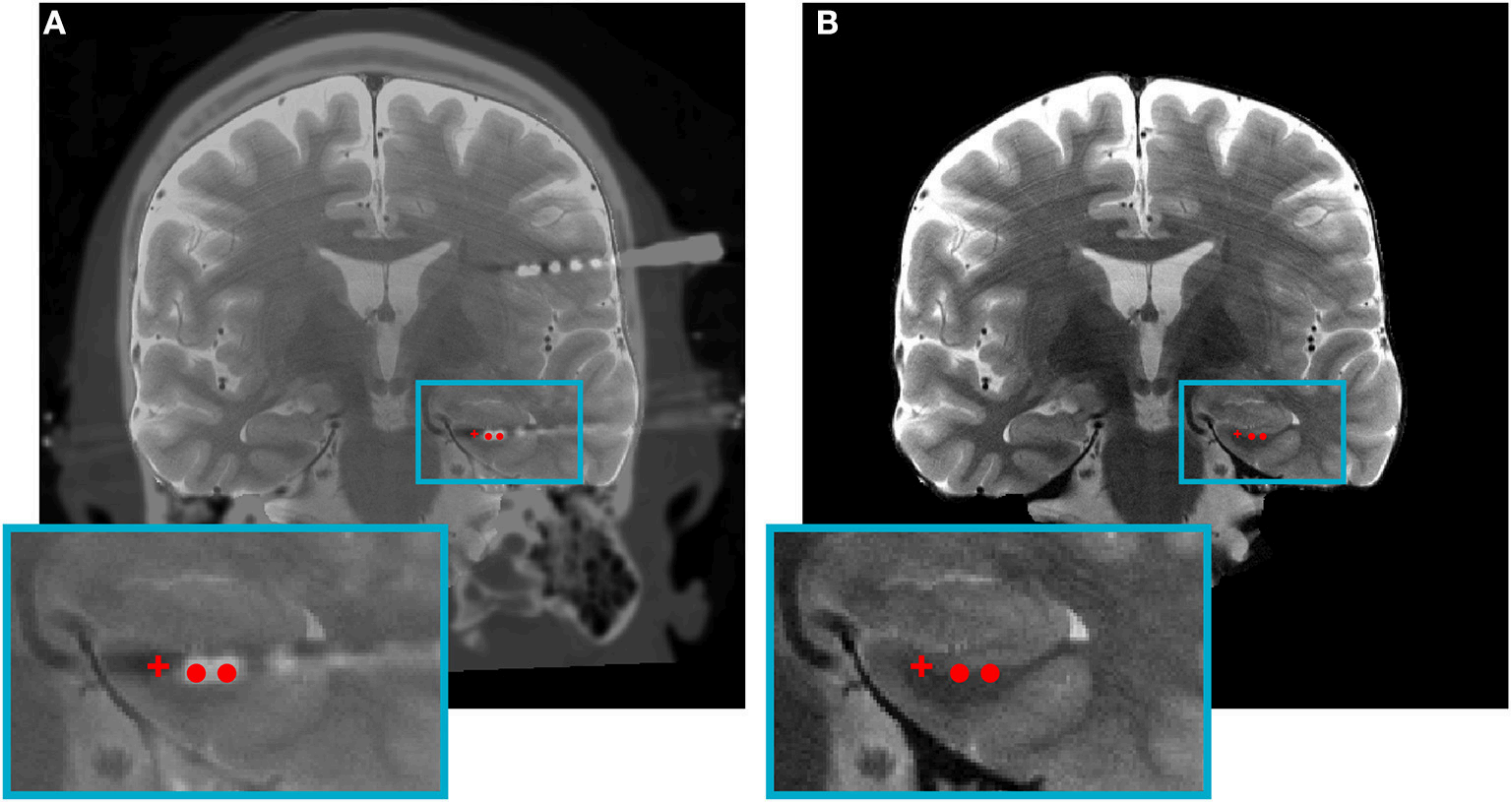
An example image for studies to follow when reporting single participant electrode localizations for bipolar macro-stimulation or monopolar micro-stimulation. **(A)** Coronal view of a co-registered image of a pre-implantation high-resolution CT and post-implantation high-resolution MRI with overlaid electrode contact segmentations. **(B)** The outcome of the electrode localization procedure where individual electrode contact locations within the left hippocampus are overlaid onto a high-resolution MRI for final visualization and reporting. Red circles: bipolar macro-stimulation contacts; red crosshair: monopolar microstimulation contact.

Studies should include, at the minimum, an electrode localization figure for an example participant in the main analyses (e.g., Figure 1) and an electrode localization figure or table showing the placement of electrode contacts for each participant, perhaps in the Supplemental Materials. Each participant's electrode localizations should be included with a unique subject ID that is consistent throughout the manuscript, allowing localization information to be cross-referenced to other information (e.g., behavioral or electrophysiological results) regarding individual subjects. If stimulation or electrophysiological analyses are done using bipolar montages, localizations should be reported for each individual electrode contact. Since accurate localization of electrodes depends on the quality of both the post-implantation (e.g., CT) scans and pre-implantation (e.g., MRI) scans, studies should report acquisition parameters, and consequent voxel resolution of all scans to enable accurate evaluation and future replication. Furthermore, in addition to detailed description of registration procedures, the known minimal error associated with the procedure should be reported. Subdural electrodes provide an additional challenge for localizations, as brain shift frequently accompanies the implantation procedure. Thus, studies should either correct for that shift or acquire high resolution post-implantation MRI scans (Groppe et al., 2017).

Several toolboxes are available for demarcating subcortical anatomy in individual subjects to aid in visualization of small subregions in which electrode contacts reside (e.g., Yushkevich et al., 2010). The toolbox used and any relevant parameter settings should be reported. Given the differences between the electrical properties of gray and white matter, and because electrodes may not lie neatly inside a single tissue type, it is helpful to report an estimation of relative amount of gray and white matter in the vicinity of each stimulating electrode (Mercier et al., 2017).

Electrode locations visualized on an average template brain could also be included to demonstrate patterns of electrode distribution across participants using known toolboxes (e.g., Xia et al., 2013). However, non-linear registration and inter-subject anatomical variability can cause aggregate figures to lose valuable within-subject detail that may help explain variability in findings across the sample and across studies from different surgical sites. With areas such as the MTL, where subregions can be millimeters in thickness, these subtle differences could be detrimental to replication efforts if individual localization information is not presented as well. Therefore, group brain visualizations of electrodes should not be the only method of electrode localization but rather supplemental to individual subject electrode localizations. Furthermore, registration procedures from individual to group or template brain images must be reported in detail.

Some studies include participants with different types of electrodes with varying diameter and/or spacing between contacts. For example, subdural electrodes can have up to 10 mm of space between adjacent stimulating bipolar contacts. Depth electrodes, which penetrate the MTL can vary, usually with 3–10 mm between contacts. Given the large spacing between electrode contacts used for bipolar stimulation, a pair of stimulating contacts rarely falls within the same area. Most studies, nonetheless, refer to one electrode location in a single brain area (e.g., only one contact or the calculated midpoint between two bipolar contacts), but it is not always clear how the reported area was chosen. We recommend reporting the location of each contact when bipolar stimulation is used. Indeed, providing this information could aid tremendously in the comparison of research studies and replication of methodologies across sites.

## Describe Behavioral Task Design

Studies should report in detail the behavioral task design and why it was selected. Ideally, enough details should be reported such that independent researcher would be able to replicate the task. Studies should also report if there were any differences in the behavioral design across subjects such as difficulty level (e.g., number of stimuli presented) and if so, account for this variable in their statistical analyses. Details of any unique circumstances for a given participant should be reported. How behavioral performance was calculated should be explained in detail and raw performance metrics for each condition should be reported—ideally for each individual subject—not only changes in performance between conditions (e.g., stimulation vs. non-stimulation).

## Report All Parameters of Stimulation

The electric field generated by intracranial stimulation electrodes is thought to govern the neural response to stimulation (Butson et al., 2007). As such, there are many variables that can modify the generated electric field and, thus, lead to different electrophysiological and behavioral outcomes.

### Amplitude and Impedance

The amplitude of stimulation is controlled either in voltage or current domain, and the domain chosen should be reported. Although voltage is a critical factor, in particular, for determining the volume of tissue affected (Butson et al., 2007), current-controlled stimulation protocols have been implemented recently (Preda et al., 2016). Whether there are differences in clinical outcomes or therapeutic advantages of each type of stimulation is debated (Bronstein et al., 2015; York and Moro, 2017). Impedance of the stimulating electrode should also be measured and reported. This is important for participant safety reasons, but additionally, the reporting of impedance in combination with either voltage or current allows for translating to the other domain, thus enabling meta-analyses to choose a consistent measure.

### Frequency

Varying frequencies of stimulation have been shown to affect the outcome of DBS studies in domains such as Parkinson's disease (Moreau et al., 2008), dystonia (Kupsch et al., 2003), and depression (Mayberg et al., 2005). No intracranial study to date has systematically investigated the relationship between different stimulation frequencies and declarative memory performance. Many studies have used continuous stimulation protocols for which reporting frequency and amplitude is sufficient. Animal studies have shown that theta-burst stimulation is especially effective for inducing long-term potentiation (Larson et al., 1986) and could, thus, be beneficial for memory. Recently, theta-burst stimulation has been used in humans (Miller et al., 2015; Titiz et al., 2017; Inman et al., 2018; Kim et al., 2018), and in such studies frequency of bursts must be reported along with frequency within each burst.

### Charge Density

For stimulation to have an effect, it is necessary to deliver efficacious amount of charge without compromising the electrochemical balance of the tissue, which can lead to potential safety issues (Rose and Robblee, 1990). Thus, stimulation charge density is an important factor to consider, which can be calculated from the combination of the following parameters: duration of the stimulation pulse (T); surface area of the stimulation electrode contact (or contacts if bipolar stimulation, A); and the amplitude (current) of the pulse (I), according to the following equation: $\rho_{Q}=\frac{IT}{A}$.

In order to facilitate future replication and meta-analyses, all numbers related to the waveform of the stimulation pulse and charge density should be reported.

In addition, reports should include any unique patient-level effects; such as whether they were aware of stimulation or whether there were any consequent side effects stimulation that was unexpected.

## Detail Timing of Stimulation Relative to Memory Task

The temporal specificity of stimulation, both in terms of its duration and its timing with respect to other behavioral task parameters, can introduce variability in study outcomes. For instance, stimulation applied in the hippocampus at encoding but not retrieval impairs memory (Lacruz et al., 2010). Additionally, applying intermittent stimulation in the nucleus basalis in adult monkeys enhances working memory, but continuous stimulation leads to memory impairment (Liu et al., 2017). Studies should, therefore, report the timing of stimulation relative to stimulus presentation and whether stimulation was applied during encoding, distraction, retrieval, or any other time periods.

## Choose the Appropriate Statistical Model

Statistical model assumptions commonly violated in intracranial stimulation studies of declarative memory are the assumption of independence of observations and assumptions regarding the distribution of the outcome or the errors. Although studies generally use repeated measures designs, not all statistical analyses address the non-independence of observations inherent in these designs. Many reported statistical models assume independence of observations but have nonetheless included observations from the same participant. Violation of the independence assumption can result in biased parameter estimates, underestimated variability of model parameters, and overly optimistic *p*-values (Hox et al., 2017). Researchers should consider using methods that support estimation assuming non-independence within clusters of observations, such as mixed models. Mixed models can include random effects that simultaneously attempt to account for the non-independence within clusters and also quantify heterogeneity across clusters. Many standard regression models (e.g., linear, logistic) have been extended to mixed models to allow for random effects. Parametric statistical models (e.g., *t*-tests, ANOVA, regressions) make assumptions about the distributions of the dependent variables or the errors, the individual deviations from the population means. While classical ANOVA and linear regression assume normally distributed errors, logistic regression assumes that the outcome is binomially-distributed, appropriate for a success/failure variable. Logistic regression can be used to model the probability of successfully recalling a single item, and to estimate effects of predictors that vary at the item level (e.g., stimulation on/off condition, time since the last stimulation). Additionally, logistic regression accounts for differing numbers of trials across participants, which linear regression of aggregated mean probabilities cannot. Finally, logistic regression can be extended to mixed models including random effects to address non-independence of success/failure outcomes within clusters of observations.

## Transparently Report Details of the Statistical Analysis

Given the rarity of intracranial stimulation studies of declarative memory, transparency in reporting of all statistical analyses is crucially needed. Unfortunately, however, relevant details are often obscured with only the statistical model used and *p*-values reported. Below we outline three details that should be clearly specified to improve reader evaluation of the statistical analyses including (1) sample sizes, (2) effect sizes, and (3) the number of hypotheses tested and *p*-values estimated over the entirety of the statistical analysis. Omission of these details can result in improper evaluation and interpretation of findings and problems with study replication.

### Sample Sizes

Sample sizes inform the reader about the replicability of the analysis and should be reported for each statistical analysis or model. Sample sizes can vary greatly within the same study but across analyses when subjects are used selectively in analyses or when some analyses use aggregated variables (e.g., means, sums). Intracranial stimulation studies of declarative memory commonly use a repeated-measures design where participants experience several encoding sessions, brain regions, and memory tests. While some studies have modeled the probability of recall of a single item (Ezzyat et al., 2018; Kim et al., 2018), others have modeled the mean probability of recall of items aggregated at the level of session (Jacobs et al., 2016), brain location (Jacobs et al., 2016; Merkow et al., 2017), or even participant (Kucewicz et al., 2018a), at times within the same study. The burden on the reader to remember the sample sizes at each level of aggregation can be cumbersome, so every analysis presented should include sample size information.

### Effect Sizes

Effect sizes provide an estimate of the magnitude of the effect, which significance tests and *p*-values do not. Significance tests may suggest whether observed changes in memory were likely to arise by chance, but the associated statistics do not directly estimate how large that change is and whether it is substantively meaningful. Readers should be given the opportunity to interpret effect sizes for themselves, so effect sizes should be reported and interpreted. Precision in estimation of effect sizes can be expressed with confidence intervals, giving the reader a range of effect sizes compatible with the data. Both unstandardized effect sizes (e.g., mean differences, regression coefficients), and standardized effect sizes (e.g., Cohen's *d, R*^2^), are worth reporting. The shift of emphasis away from null hypothesis testing and toward effect estimation is being advocated in many fields to address problems of low replicability (Cumming, 2014).

### Number of Hypothesis Tests and *P*-Values

Another potential source of low replicability is the practice of reporting only significant findings. A low *p*-value from a single, planned statistical model will generally be more persuasive than a single low *p*-value from 10 modifications of a statistical model. However, researchers generally present the final model as if it were the only one tested. As more hypotheses are tested, the probability of making an erroneous inference increases, and without accounting for the additional testing, the reader's interpretation of reported *p*-values will generally be too optimistic. To combat misinterpretation of *p*-values, the American Statistical Association recommends reporting the number of hypotheses and all statistical analyses conducted throughout the study (American Statistical Association, 2016). Additionally, researchers should make appropriate adjustments to significance thresholds to account for multiple comparisons.

## Electrophysiology

Oftentimes, in addition to the implanted stimulation electrode—which typically cannot record neural signals during stimulation or at all—additional electrode contacts nearby or in other brain areas capable of recording electrophysiological activity are present. This situation presents additional opportunities to analyze neural responses during the behavioral task in response to sensory input, as well as to evaluate the effects of stimulation on those responses. In this case, the locations of both the stimulating and recording electrodes should be reported following the guidelines detailed above.

It is outside the scope of this review to give thorough guidelines for reporting on electrophysiology, however the presence of stimulation artifact in recorded data must be dealt with prior to drawing conclusions. It is critical to report the method used for rejecting stimulation artifacts, and include figures of signals before and after data cleaning procedures (e.g., Basir-Kazeruni et al., 2017; O'Shea and Shenoy, 2018). Because many stimulation artifact rejection algorithms rely on the assumption that the artifact does not saturate the signal (Basir-Kazeruni et al., 2017; O'Shea and Shenoy, 2018), amplitude of the artifact and recording ranges should be reported.

In the case where electrophysiological recordings are conducted in subjects with epilepsy, the ability to record also allows for detailed analysis of epileptic discharges during the memory task. These epileptic spikes have been shown to interfere with memory and other cognitive performance (Kleen et al., 2013; Ung et al., 2017) and should be taken into account when analyzing the data. In particular, manuscripts should report how epileptic discharges were detected and how much data was affected and/or excluded based on these detections. Statistical analyses of main effects should take into account the prevalence and timing of interictal spikes and summary statistics of these variables should be included.

## Closed Loop Stimulation

Most studies to date have been conducted using open-loop stimulation, which considers only external factors in determining when and whether to stimulate. However, increasingly, studies are able to record neural signals during behavioral tasks and then use internal factors of the neural state to determine the precise timing of stimulation, commonly referred to as closed-loop stimulation. Internal brain states can be used to determine the timing of stimulation [e.g., at a particular phase of an endogenous neural oscillation (Fell et al., 2013)] or to make a decision about whether or not to stimulate at all.

The challenge of closed-loop decision-making has been recently tackled using machine learning models: a set of stimulation-free trials with neural data and labels indicating subsequent memory performance was collected and used to train a model to recognize features that predict memory states for a particular participant, which in turn could drive stimulation decisions (Ezzyat et al., 2017, 2018).

It is important when reporting closed-loop stimulation studies to not only report all of the parameters required for open-loop stimulation, but also include detailed descriptions of the prediction model used to trigger stimulation, how the training set was collected and size of the training set, model performance [e.g., area under the receiver operant characteristic curve (AUC)], criterion for when stimulation was given/withheld, and elapsed time between collecting training data and testing closed-loop stimulation. Ideally, to demonstrate that the model produces relevant decisions at the time of an experiment, closed-loop experiments should include both test, and control trials that are collected within a single experimental session. In both types of trials, the model's stimulation decision would be recorded, however stimulation would only be applied during the test trials. This would allow for gauging the model performance without the confound of the stimulation.

## Conclusions

Intracranial stimulation studies of declarative memory have reported both enhancement and impairment of memory, yet the specific factors that give rise to these differences in memory modulation are unclear. What is evident, however, are numerous differences across study sites in methodologies, including but not limited to details of participants, behavioral task design, electrode characteristics, electrode placements, stimulation parameters, timing of stimulation, and statistical methods. While true replication may be difficult in this field due to the rarity of participants and difficulties of completing large sample studies that are consistent in various within-sample characteristics, the first step toward replication should be transparent reporting of methods and results. We therefore, propose a set of guidelines to reporting and issues to consider when completing future intracranial stimulation studies of declarative memory.

## Author Contributions

NS, ZM, EM, and AL wrote manuscript. NS, ZM, and EM contributed to the figure and table.

### Conflict of Interest Statement

The authors declare that the research was conducted in the absence of any commercial or financial relationships that could be construed as a potential conflict of interest.

## References

[B1] American Statistical Association (2016). Statement on statistical significance and P-values. Am. Stat. 70, 129–133. 10.1080/00031305.2016

[B2] Basir-Kazeruni S. Vlaski S. Salami H. Sayed A. H. Marković D. (2017). A blind adaptive stimulation artifact rejection (ASAR) engine for closed-loop implantable neuromodulation systems, in 2017 8th International IEEE/EMBS Conference on Neural Engineering (NER) (Shanghai), 186–189.

[B3] Benabid A. L. Pollak P. Louveau A. Henry S. de Rougemont J. (1987). Combined (thalamotomy and stimulation) stereotactic surgery of the VIM thalamic nucleus for bilateral Parkinson disease. Appl. Neurophysiol. 50, 344–346. 10.1159/000100803 3329873

[B4] Bronstein J. M. Tagliati M. McIntyre C. Chen R. Cheung T. Hargreaves E. L.. (2015). The rationale driving the evolution of deep brain stimulation to constant-current devices. Neuromodulation 18, 85–88; discussion 88–89. 10.1111/ner.12227 25171762

[B5] Brown P. Williams D. (2005). Basal ganglia local field potential activity: character and functional significance in the human. Clin. Neurophysiol. 116, 2510–2519. 10.1016/j.clinph.2005.05.009 16029963

[B6] Butson C. R. Cooper S. E. Henderson J. M. McIntyre C. C. (2007). Patient-specific analysis of the volume of tissue activated during deep brain stimulation. Neuroimage 34, 661–670. 10.1016/j.neuroimage.2006.09.034 17113789 PMC1794656

[B7] Cherlow D. G. Dymond A. M. Crandall P. H. Walter R. D. Serafetinides E. A. (1977). Evoked response and after-discharge thresholds to electrical stimulation in temporal lobe epileptics. Arch. Neurol. 34, 527–531. 10.1001/archneur.1977.00500210029003 889493

[B8] Coleshill S. G. Binnie C. D. Morris R. G. Alarcon G. van Emde Boas W. Velis D. N.. (2004). Material-specific recognition memory deficits elicited by unilateral hippocampal electrical stimulation. J. Neurosci. 24, 1612–1616. 10.1523/JNEUROSCI.4352-03.2004 14973245 PMC6730466

[B9] Cumming G. (2014). The new statistics:why and how. Psychol. Sci. 25, 7–29. 10.1177/0956797613504966 24220629

[B10] Delis D. C. Kramer J. H. Kaplan E. Ober B. A. (2000). California Verbal Learning Test, 2nd Edn. San Antonio, TX: Psychological Corporation.

[B11] Ezzyat Y. Kragel J. E. Burke J. F. Levy D. F. Lyalenko A. Wanda P.. (2017). Direct brain stimulation modulates encoding states and memory performance in humans. Curr. Biol. 27, 1251–1258. 10.1016/j.cub.2017.03.028 28434860 PMC8506915

[B12] Ezzyat Y. Wanda P. A. Levy D. F. Kadel A. Aka A. Pedisich I.. (2018). Closed-loop stimulation of temporal cortex rescues functional networks and improves memory. Nat. Commun. 9:365. 10.1038/s41467-017-02753-0 29410414 PMC5802791

[B13] Fell J. Staresina B. P. Do Lam A. T. Widman G. Helmstaedter C. Elger C. E.. (2013). Memory modulation by weak synchronous deep brain stimulation: a pilot study. Brain Stimul. 6, 270–273. 10.1016/j.brs.2012.08.001 22939277

[B14] Fernández G. Hufnagel A. Helmstaedter C. Zentner J. Elger C. E. (1996). Memory function during low intensity hippocampal electrical stimulation in patients with temporal lobe epilepsy. Eur. J. Neurol. 3, 335–344.

[B15] Groppe D. M. Bickel S. Dykstra A. R. Wang X. Megevand P. Mercier M. R.. (2017). iELVis: an open source MATLAB toolbox for localizing and visualizing human intracranial electrode data. J. Neurosci. Methods 281, 40–48. 10.1016/j.jneumeth.2017.01.022 28192130

[B16] Halgren E. Wilson C. L. (1985). Recall deficits produced by afterdischarges in the human hippocampal formation and amygdala. Electroencephalogr. Clin. Neurophysiol. 61, 375–380. 10.1016/0013-4694(85)91028-4 2412789

[B17] Halgren E. Wilson C. L. Stapleton J. M. (1985). Human medial temporal-lobe stimulation disrupts both formation and retrieval of recent memories. Brain Cogn. 4, 287–295. 10.1016/0278-2626(85)90022-3 4027062

[B18] Hamani C. McAndrews M. P. Cohn M. Oh M. Zumsteg D. Shapiro C. M.. (2008). Memory enhancement induced by hypothalamic/fornix deep brain stimulation. Ann. Neurol. 63, 119–123. 10.1002/ana.21295 18232017

[B19] Hansen N. Chaieb L. Staresina B. Hampel K. Elger C. E. Surges R. (2017). Memory encoding-related anterior hippocampal potentials are modulated by electric deep brain stimulation of the entorhinal area. Brain Stimul. 10:406 10.1016/j.brs.2017.01.205 29034573

[B20] Hox J. J. Moerbeek M. van de Schoot R. (2017). Mutilevel Analysis Techniques and Application, 3rd Edn. New York, NY: Routledge.

[B21] Inman C. S. Manns J. R. Bijanki K. R. Bass D. I. Hamann S. Drane D. L.. (2018). Direct electrical stimulation of the amygdala enhances declarative memory in humans. Proc. Natl. Acad. Sci. U.S.A. 115, 98–103. 10.1073/pnas.1714058114 29255054 PMC5776809

[B22] Jacobs J. Miller J. Lee S. A. Coffey T. Watrous A. J. Sperling M. R.. (2016). Direct electrical stimulation of the human entorhinal region and hippocampus impairs memory. Neuron 92, 983–990. 10.1016/j.neuron.2016.10.062 27930911

[B23] Kesner R. P. (1982). Brain stimulation: effects on memory. Behav. Neural. Biol. 36, 315–367. 10.1016/S0163-1047(82)90762-2 6135412

[B24] Kim K. Ekstrom A. D. Tandon N. (2016). A network approach for modulating memory processes via direct and indirect brain stimulation: toward a causal approach for the neural basis of memory. Neurobiol. Learn. Mem. 134, 162–177. 10.1016/j.nlm.2016.04.001 27066987

[B25] Kim K. Schedlbauer A. Rollo M. Karunakaran S. Ekstrom A. D. Tandon N. (2018). Network-based brain stimulation selectively impairs spatial retrieval. Brain Stimul. 11, 213–221. 10.1016/j.brs.2017.09.016 29042188 PMC5729089

[B26] Kleen J. K. Scott R. C. Holmes G. L. Roberts D. W. Rundle M. M. Testorf M.. (2013). Hippocampal interictal epileptiform activity disrupts cognition in humans. Neurology 81, 18–24. 10.1212/WNL.0b013e318297ee50 23685931 PMC3770206

[B27] Koubeissi M. Z. Kahriman E. Syed T. U. Miller J. Durand D. M. (2013). Low-frequency electrical stimulation of a fiber tract in temporal lobe epilepsy. Ann. Neurol. 74, 223–231. 10.1002/ana.23915 23613463

[B28] Kucewicz M. T. Berry B. M. Kremen V. (2018a). Electrical stimulation modulates high γ activity and human memory performance. eNeuro 5:ENEURO.0369-17.2018. 10.1523/ENEURO.0369-17.2018 29404403 PMC5797477

[B29] Kucewicz M. T. Berry B. M. Miller L. R. Khadjevand F. Ezzyat Y. Stein J. M.. (2018b). Evidence for verbal memory enhancement with electrical brain stimulation in the lateral temporal cortex. Brain 141, 971–978. 10.1093/brain/awx373 29324988

[B30] Kupsch A. Klaffke S. Kuhn A. A. Meissner W. Arnold G. Schneider G. H.. (2003). The effects of frequency in pallidal deep brain stimulation for primary dystonia. J. Neurol. 250, 1201–1205. 10.1007/s00415-003-0179-0 14586602

[B31] Lacruz M. E. Valentin A. Seoane J. J. Morris R. G. Selway R. P. Alarcon G. (2010). Single pulse electrical stimulation of the hippocampus is sufficient to impair human episodic memory. Neuroscience 170, 623–632. 10.1016/j.neuroscience.2010.06.042 20643192

[B32] Larson J. Wong D. Lynch G. (1986). Patterned stimulation at the theta frequency is optimal for the induction of hippocampal long-term potentiation. Brain Res. 368, 347–350. 10.1016/0006-8993(86)90579-2 3697730

[B33] Liu R. Crawford J. Callahan P. M. Terry A. V. Jr. Constantinidis C. Blake D. T. (2017). Intermittent stimulation of the nucleus basalis of meynert improves working memory in adult monkeys. Curr. Biol. 27, 2640–2646.e2644. 10.1016/j.cub.2017.07.021 28823679 PMC5759307

[B34] Lozano A. M. Fosdick L. Chakravarty M. M. Leoutsakos J. M. Munro C. Oh E.. (2016). A phase II study of fornix deep brain stimulation in mild Alzheimer's disease. J. Alzheimers Dis. 54, 777–787. 10.3233/JAD-160017 27567810 PMC5026133

[B35] Mayberg H. S. Lozano A. M. Voon V. McNeely H. E. Seminowicz D. Hamani C.. (2005). Deep brain stimulation for treatment-resistant depression. Neuron 45, 651–660. 10.1016/j.neuron.2005.02.014 15748841

[B36] Mercier M. R. Bickel S. Megevand P. Groppe D. M. Schroeder C. E. Mehta A. D.. (2017). Evaluation of cortical local field potential diffusion in stereotactic electro-encephalography recordings: a glimpse on white matter signal. Neuroimage 147, 219–232. 10.1016/j.neuroimage.2016.08.037 27554533

[B37] Merkow M. B. Burke J. F. Ramayya A. G. Sharan A. D. Sperling M. R. Kahana M. J. (2017). Stimulation of the human medial temporal lobe between learning and recall selectively enhances forgetting. Brain Stimul. 10, 645–650. 10.1016/j.brs.2016.12.011 28073638 PMC5410394

[B38] Meyers J. E. Meyers K. R. (1995). Rey Complex Figure Test and Recognition Trial. Odessa, FL: Psychological Assessment Resources.

[B39] Miller J. P. Sweet J. A. Bailey C. M. Munyon C. N. Luders H. O. Fastenau P. S. (2015). Visual-spatial memory may be enhanced with theta burst deep brain stimulation of the fornix: a preliminary investigation with four cases. Brain 138(Pt 7), 1833–1842. 10.1093/brain/awv095 26106097

[B40] Moreau C. Defebvre L. Destee A. Bleuse S. Clement F. Blatt J. L.. (2008). STN-DBS frequency effects on freezing of gait in advanced Parkinson disease. Neurology 71, 80–84. 10.1212/01.wnl.0000303972.16279.46 18420482

[B41] Nuttin B. Cosyns P. Demeulemeester H. Gybels J. Meyerson B. (1999). Electrical stimulation in anterior limbs of internal capsules in patients with obsessive-compulsive disorder. Lancet 354:1526. 10551504 10.1016/S0140-6736(99)02376-4

[B42] O'Shea D. J. Shenoy K. V. (2018). ERAASR: an algorithm for removing electrical stimulation artifacts from multielectrode array recordings. J. Neural. Eng. 15:026020. 10.1088/1741-2552/aaa365 29265009 PMC5833982

[B43] Poldrack R. A. Fletcher P. C. Henson R. N. Worsley K. J. Brett M. Nichols T. E. (2008). Guidelines for reporting an fMRI study. Neuroimage 40, 409–414. 10.1016/j.neuroimage.2007.11.048 18191585 PMC2287206

[B44] Pouratian N. Zheng Z. Bari A. A. Behnke E. Elias W. J. Desalles A. A. (2011). Multi-institutional evaluation of deep brain stimulation targeting using probabilistic connectivity-based thalamic segmentation. J. Neurosurg. 115, 995–1004. 10.3171/2011.7.JNS11250 21854118

[B45] Preda F. Cavandoli C. Lettieri C. Pilleri M. Antonini A. Eleopra R.. (2016). Switching from constant voltage to constant current in deep brain stimulation: a multicenter experience of mixed implants for movement disorders. Eur. J. Neurol. 23, 190–195. 10.1111/ene.12835 26498428

[B46] Ressler K. J. Mayberg H. S. (2007). Targeting abnormal neural circuits in mood and anxiety disorders: from the laboratory to the clinic. Nat. Neurosci. 10, 1116–1124. 10.1038/nn1944 17726478 PMC2444035

[B47] Riva-Posse P. Choi K. S. Holtzheimer P. E. McIntyre C. C. Gross R. E. Chaturvedi A.. (2014). Defining critical white matter pathways mediating successful subcallosal cingulate deep brain stimulation for treatment-resistant depression. Biol. Psychiatry 76, 963–969. 10.1016/j.biopsych.2014.03.029 24832866 PMC4487804

[B48] Rose T. L. Robblee L. S. (1990). Electrical stimulation with Pt electrodes. VIII. Electrochemically safe charge injection limits with 0.2 ms pulses. IEEE Trans. Biomed. Eng. 37, 1118–1120. 10.1109/10.61038 2276759

[B49] Schulze-Bonhage A. (2017). Brain stimulation as a neuromodulatory epilepsy therapy. Seizure 44, 169–175. 10.1016/j.seizure.2016.10.026 27876408

[B50] Sreekumar V. Wittig J. H. Jr. Sheehan T. C. Zaghloul K. A. (2017). Principled approaches to direct brain stimulation for cognitive enhancement. Front. Neurosci. 11:650. 10.3389/fnins.2017.00650 29249927 PMC5714894

[B51] Suthana N. Fried I. (2014). Deep brain stimulation for enhancement of learning and memory. Neuroimage 85, 996–1002. 10.1016/j.neuroimage.2013.07.066 23921099 PMC4445933

[B52] Suthana N. Haneef Z. Stern J. Mukamel R. Behnke E. Knowlton B.. (2012). Memory enhancement and deep-brain stimulation of the entorhinal area. N. Engl. J. Med. 366, 502–510. 10.1056/NEJMoa1107212 22316444 PMC3447081

[B53] Titiz A. S. Hill M. R. H. Mankin E. A. M Aghajan Z. Eliashiv D. Tchemodanov N.. (2017). Theta-burst microstimulation in the human entorhinal area improves memory specificity. Elife 6:29515. 10.7554/eLife.29515 29063831 PMC5655155

[B54] Ung H. Cazares C. Nanivadekar A. Kini L. Wagenaar J. Becker D.. (2017). Interictal epileptiform activity outside the seizure onset zone impacts cognition. Brain 140, 2157–2168. 10.1093/brain/awx143 28666338 PMC6167607

[B55] Vidailhet M. Vercueil L. Houeto J. L. Krystkowiak P. Benabid A. L. Cornu P.. (2005). Bilateral deep-brain stimulation of the globus pallidus in primary generalized dystonia. N. Engl. J. Med. 352, 459–467. 10.1056/NEJMoa042187 15689584

[B56] Wechsler D. (2005). Wechsler Memory Scale, Revised. New York, NY: Psychological Corporation/ Harcourt Brace Jovanovich.

[B57] Xia M. Wang J. He Y. (2013). BrainNet viewer: a network visualization tool for human brain connectomics. PLoS ONE 8:e68910. 10.1371/journal.pone.0068910 23861951 PMC3701683

[B58] York M. K. Moro E. (2017). No differences in neuropsychological outcomes between constant current and voltage current subthalamic deep brain stimulation for Parkinson's disease. Ann. Transl. Med. 5:177. 10.21037/atm.2017.03.43 28480213 PMC5401669

[B59] Yushkevich P. A. Wang H. Pluta J. Das S. R. Craige C. Avants B. B.. (2010). Nearly automatic segmentation of hippocampal subfields in *in vivo* focal T2-weighted MRI. Neuroimage 53, 1208–1224. 10.1016/j.neuroimage.2010.06.040 20600984 PMC2939190

