# Supplementary material for: Reporting Guidelines and Issues to Consider for Using Intracranial Brain Stimulation in Studies of Human Declarative Memory
Source: Front Neurosci. 2018 Dec 4;12:905. doi: 10.3389/fnins.2018.00905 (PMC6288473; doi:10.3389/fnins.2018.00905)
Supplement: Supplementary file 1 [file Data_Sheet_1.docx]

**Appendix A. Guidelines for reporting (Checklist)**

For any parameter below that varied between participants, it is crucial to report the parameter value for each participant, along with a description of how these parameters were determined.

**Human participants**

Demographics (e.g., Age, Handedness, Gender)

Neuropsychological scores (including any memory related scores and, if relevant, language dominance)

MRI abnormalities (e.g., left hippocampal sclerosis)

Determined seizure onset areas

Medication taken (e.g., antiepileptic drugs)

Comorbid disorders if any (e.g., depression)

Seizure history (e.g., number and time of seizures relative to testing session)

Any previous neurosurgical procedures

**Electrodes and localization**

Images acquired and timing (e.g., pre-implantation MRI and post-implantation CT)

Image registration techniques and software used

Type of surgical approach used (e.g., temporolateral or transoccipital)

Type of electrode used (e.g., strip, grid, depth), diameter and length of contacts, and spacing between contacts (e.g., 3 mm or 10 mm center to center)

Electrodes used for stimulation versus recording in each separate analysis

Method for defining brain regions with localized electrodes (e.g., automatic segmentation software, manual segmentation using which protocol, or by visual inspection)

Electrode location MRIs for each participant (at least for the subgroup that shows main effect) and a group map of electrodes registered to a common space

**Stimulation parameters**

Current, impedance, voltage, charge density, frequency, pulse width, train duration

Bipolar or monopolar stimulation

Hemisphere stimulated (i.e., left, right, bilateral)

Timing of stimulation relative to behavioral task trials for each session (e.g., prior to trial onset, during or after)

Distribution of stimulation during the task (randomized or alternating) and counterbalancing method

**Behavioral task**

Number of blocks, sessions or trials

Duration of trials, blocks and intertrial intervals

Detailed descriptions of distractor or non-mnemonic control tasks

Task instructions given to participant

Stimuli used and modality of presentation (visual, auditory, etc.)

Number of stimuli (If difficulty was tailored for each individual participant, report method of how this was determined)

Were new or repeated stimuli used across trials, blocks, or sessions?

**Behavioral performance**

Details of calculation of performance metric, including any normalization applied

If publishing multiple studies from a common dataset include, at the minimum, the identical performance measurements to enable comparison across studies

Report raw individual performance data values not just differences between conditions (e.g., stimulation versus non-stimulation)

**Data acquisition**

Electrophysiological recording system used and sampling rate

Stimulator used and how it was triggered (manual or automatic)

If closed loop stimulation used, how was it triggered and when was it delivered relative to task trial?

**Safety and Ethics**

IRB and/or FDA approval

Safety precautions taken

Seizures or after-discharges elicited

Participant reports of awareness of stimulation or other effects?

**Electrophysiological analyses**

Was there stimulation artifact present and if so how was this data analyzed?

Was there any seizure activity or after discharges and if so how was this dealt with in analyses?

If the data (e.g., power, amplitude, etc.) is normalized, is the normalization procedure clearly described?

If machine learning models were used to analyze the data, include information on; the type of model used; distribution of training, testing, and cross-validation (if any) data sets; how over-fitting and feature selection were handled; measures of model performance (e.g., AUC, confusion matrix, MSE, etc.)

**Statistical analyses**

Choose a statistical model appropriate for the test hypothesis and the study design used to collect the data.

Evaluate plausibility of model assumptions, paying special attention to distributional assumptions and the assumption of independence of observations, which is violated in repeated-measures designs.

Describe all statistical analyses conducted, identifying the statistical model/test used, dependent variables, and independent variables, detailing transformations of any variables (e.g., aggregation, logarithms, squaring).

Include relevant participant and environment variables (e.g., medication, SOZ, tissue abnormality) in statistical model to control for confounding with experimental manipulations

Report samples sizes for each analysis, with specific sizes for each condition, testing session or brain region tested (e.g., “10 participants received stimulation in the hippocampus during the encoding period of the task”).

Effect sizes (e.g., mean differences, regression coefficients, Cohen’s *d*, or *R^2^*) and measures of their variability (confidence intervals or standard errors) should be reported in addition to or instead of p-values.

For statistical analyses conducted during the study but not described in the manuscript, report the number of hypothesis tested and p-values calculated. Adjust significance thresholds for multiple hypotheses to control rate of erroneous inferences (e.g., family-wise error rate, false discovery rate).

**Figures and Tables**

From which participant does the example figure come (use the same participant ID that is reported in the participant information table)?

If statistically significant results are shown, include sample size and what they refer to (i.e., number of electrodes, testing sessions, participants)? If multiple sample sizes are relevant then report all of them (e.g., N = 10 participants, M = 16 electrode locations, n = 24 testing sessions).
